# Efficacy of internal fixation with mini plate and internal fixation with hollow screw for Regan-Morrey type II and III ulna coronoid fractures

**DOI:** 10.1186/s12891-018-2117-4

**Published:** 2018-06-19

**Authors:** Hong-Wei Chen, Hong-Hui He, Bin-Li Gao

**Affiliations:** 1 Department of Orthopedic Surgery, Yiwu Central Hospital, the Affiliated Yiwu Hospital of Wenzhou Medical University, Yiwu, 322000 People’s Republic of China; 2 Department of Joint Surgery and Sports Medicine, the Affiliated Nanhua Hospital, University of South China, Hengyang, 421002 People’s Republic of China; 3 Department of Orthopedics, the Affiliated Hospital of Inner Mongolia Medical University, No. 1, Tongdao North Road, Hohhot, 010050 The Inner Mongolia Autonomous Region People’s Republic of China

**Keywords:** Ulna coronoid fracture, Regan-Morrey type II and III, Mini plate; hollow screw, Kirschner wire, Steel wire suture, Elbow joint function, VAS pain score

## Abstract

**Background:**

Ulna coronoid fracture is a complicated injury and occurred in the coronal plane. Undeniably, there is no universally accepted approach for treating ulna coronoid fractures. Therefore, this study aimed at exploring the efficacy of different surgical treatments for Regan-Morrey type II and III ulna coronoid fractures.

**Methods:**

A total of 164 patients with ulna coronoid fractures were admitted and treated in department of orthopedics at Yiwu Central Hospital, the Affiliated Yiwu Hospital of Wenzhou Medical University for retrospective analysis. The baseline features (age, gender, time from injury to surgery and so on) before the surgery and different conditions during the surgery were compared. Following that, the Visual Analogue Scale (VAS) pain score was employed to evaluate the severity of preoperative and postoperative pain experienced by the patients in each group. Afterwards, Broberg and Morrey elbow score was used to evaluate elbow joint function and surgical effect of the patients. Lastly, the postoperative recovery and complications were compared.

**Results:**

It was firstly observed that internal fixation with mini plate and hollow screw compelled to lower average operation time and blood loss than Kirschner wire and steel wire suture. Next, the severity of postoperative pain was lessened in comparison with preoperative pain. Afterwards, mini plate and hollow screw improved elbow joint function more notable than Kirschner wire and steel wire suture, and Kirschner wire and steel wire suture resulted in higher incidence of complications and worse postoperative recovery.

**Conclusion:**

Collectively, this study clarified that for the treatment of Regan-Morrey type II and III ulna coronoid fractures, internal fixation with mini plate and hollow screw has an overall superior surgical effect than internal fixation with Kirschner wire and steel wire suture.

## Background

Coronoid fractures are seldom isolated injuries and usually associated with other elbow injuries, which serve as three major instability patterns, including type I fractures (related to terrible triad injuries), type II fractures (related to varus posteromedial rotatory instability) and type III fractures (related to transolecranon fracture-dislocations) [1]. Ulna coronoid fractures play pivotal roles in providing ulnohumeral joint stability and are regarded as stabilizers against axial, varus, posteromedial, and posterolateral forces (articular laxity was noticed in the coronal or sagittal planes) [2]. The ulna coronoid fractures are important for maintaining the stability of elbows after dislocation and can be fractured, consequently leading to a coronoid fracture, an infrequent injury occurring in about 2–15% of patients with dislocated elbows [3]. A relation between coronoid fractures with specific patterns of traumatic elbow instability has been reported [4]. Recent evidence has shown that coronoid fractures result from avulsing force of the brachialis muscle with a hyperextended position of the elbow joint, as well as shearing force acting upon the surface of the trochlear with posterior dislocation of the elbow joint [5]. At present, coronoid fractures are usually caused by traffic accidents, with some incidence from falls, explosions, and local trauma [6, 7]. Although ulna coronoid fractures are rare, they still negatively influence and impact the functional outcome if proper treatment is not administered [8]. Therefore, it is of great clinical significance to find an effective treatment option for ulna coronoid fractures.

Surgical treatment with open reduction and internal fixation has gained popularity recently [9]. A previous study supports surgical treatment of patients with elbow dislocations related to Regan-Morrey type II and III coronoid fractures for improved elbow reduction, stability, and early motion [10]. Another study suggested that rigid internal fixation could aid in treating significant displacement of coronoid process [11]. Besides, a recent study indicates that ulna coronoid fractures can be treated by internal fixation with frame shape plate and open reduction through anterior approach of the elbow, as it is an effective treatment option with minimal damage and strong fixation [12]. It has been previously reported that ulna coronoid fractures treated with internal fixation had better mechanical resistance than suture anchors for fixing small fragments and internal fixation with tension band wiring with steel wire is an excellent approach to treat coronoid fractures [13–15]. Internal fixation with mini plate is effective for early functional exercise as it provides sufficient stability and improves functional outcome [16]. Iannuzzi NP with 10 pairs of fresh frozen Regan-Morrey type II coronoid fractures cadaveric elbow found that screw fixation provided greater overall foundation with superior strength (405 N VS 207 N) and stiffness (284 thousand PA/mm VS 119 thousand PA/mm) compared to suture lasso fixation, and for the treatment of Regan-Morrey type II coronoid fractures, biomechanical property of screw fixation was superior than the property of suture lasso fixation [17]. Therefore, this study aims to compare the efficacy of internal fixation with mini plate, hollow screw, Kirschner wire and steel wire suture for the treatment of Regan-Morrey type II and III ulna coronoid fractures.

## Methods

### Ethics statement

The study was conducted with approval and under supervision of the Ethics Committee of Yiwu Central Hospital, the Affiliated Yiwu Hospital of Wenzhou Medical University. All subjects in this study were informed and all participants and their families signed informed consent in accordance with *Helsinki Declaration*. This experiment has been registered in the Chinese Clinical Laboratory Center.

### Study subjects

With the aim of retrospective analysis, a total of 164 patients (male = 90; female = 74; average age = 36.2 ± 6.7) with ulna coronoid fractures were admitted and treated in department of orthopedics at Yiwu Central Hospital, the Affiliated Yiwu Hospital of Wenzhou Medical University from September 2012 to September 2016. Among these patients, there were 102 cases of falls and 62 cases of traffic injuries; 83 cases were of left side injuries while the remaining 81 cases were of right side injuries. The reported average time from injury to operation was 4.6 ± 1.2 days. According to the different internal fixation methods, the patients were grouped into four groups for retrospective analysis: mini plate group (31 cases), hollow screw group (44 cases), Kirschner wire group (62 cases), and steel wire suture group (27 cases). Inclusion criteria was as follows: (1) all patients were diagnosed with ulnar coronoid fractures by X-ray or CT examination and according to Regan-Morrey classification and there were 101 cases of type II coronoid fractures and 63 cases of type III coronoid fractures [18]; (2) all patients had closed fractures, and the operation was performed within 1 week after injury; (3) patients with in good physical condition for surgery; (4) patients can be trained and re-examined regularly after surgery. Exclusion criteria was as follows: (1) the injured elbow had been surgically operated upon once; (2) patients with old fracture, open fracture, comminuted fractures and their free articular fragments cannot be reset with a minimally invasive method; (3) patients with elbow joint dysfunction; (4) patients with significant nerve and vascular injury interfering with postoperative rehabilitation.

### Surgical treatment

All patients were treated with routine auxiliary examination before surgery. The patients in supine position were administered a combination of intravenous inhaled anesthesia or brachial plexus anesthesia. After administering anesthesia, the C-arm X-ray machine was directed at the injured elbow and the injured elbow was adjusted to the right angle for traction reduction under observation of the C-arm X-ray machine. An anterior approach (repair of the distal biceps tendon) to the elbow joint was made. During the surgery, a longitudinal incision was made along the outside of the pronator circular muscle and a blunt tool was used to incise the subcutaneous tissue to protect the medial forearm dermatome and lateral forearm dermatome. Alongside, the circular muscles were pulled to the inside to protect the median nerve, as it was of great importance to avoid contact with the median nerve during the surgery. With the elbow joint flexed at 45°, the humeral muscle stop point and anterior elbow joint capsule as well as the ulna coronoid fractures were exposed. After operating upon the fracture, the coronoid was fixed with a mini plate, hollow screw, Kirschner wire and steel wire suture, respectively. Vital signs of the patients were monitored routinely after surgery and the patients were administered pain relief, detumescence and anti-infection treatments. After 3 days, the patients performed passive elbow flexion and elbow movement as well as active movement of wrists and fingers under the guidance of doctors.

### Outcome evaluation

The patients’ operation time and intraoperative blood loss were recorded (both the operator and the anesthetist measured intraoperative blood loss via visual method and estimated the amount of blood in the negative pressure bottle by weighing the amount of blood loss in the gauze.). The patients were followed up on 3 d, 1 month, 3 months, 6 months and 12 months after surgery (follow-up period from September 2016 to September 2017 for 1 year). Follow-up examination included X-ray (the fracture healing was evaluated by X-ray CT and the criterion for inclusion was the presence of the blurring fracture line with continuous osteophytes passing through the fracture line), fractures healing time, elbow joint activity, elbow joint stability, pain and complications. Broberg and Morrey elbow score was used to evaluate the function of elbow joint 1 year after surgery. This score is out of 100 points, with 40 points for elbow joint activity, 20 points for elbow joint strength, 5 points for elbow joint stability, and 35 points for pain; 95 ~ 100 points were regarded as excellent; 80 ~ 94 points were regarded as good; 60 ~ 79 points were regarded as medium; 0 ~ 59 points were regarded as poor [19]. Visual Analogue Scale (VAS) was used to evaluate the pain of patients 1 year after surgery (out of a score of 10 points): 0 point was no pain; 1–3 points signified mild and bearable pain; 4–6 points signified bearable pain which could affect sleep; 7–10 points signified gradually increasing and unbearable pain affecting appetite and sleep [20].

### Statistical analysis

Statistical analysis was performed using the SPSS 21.0 statistical software (IBM Corp., Armonk, NY, USA). Measurement data were presented as mean ± standard deviation. Through homogeneity of variance test, *t*-test was employed for comparing between the two groups with homogeneity of variance, and Wilcoxon rank sum test was employed for comparing among multiple groups without homogeneity of variance and normal distribution. Enumeration data were expressed by constituent ratio or rate and examined by the chi square test. A one-way analysis of variance (ANOVA) with replicate measurement was used to compare data at a specific time points within a group, while multi-group data was compared using single factor ANOVA or multi-group chi-square test. A two-sided test was performed and a value of *p* < 0.05 was considered to be statistically significant.

## Results

### Baseline characteristics of patients with ulna coronoid fractures in the four groups

Initially, the baseline characteristics of patients in each group were compared. The mini plate group (31 cases) included 9 males and 12 females with an average age of 38.8 ± 6.1 years. The hollow screw group (44 cases) included 23 males and 21 females with an average age of 35.0 ± 6.9 years. The Kirschner wire group (62 cases) included 36 males and 26 females with an average age of 35.5 ± 6.6 years. The steel wire suture group (27 cases) included 12 males and 15 females with an average age of 36.1 ± 6.4 years. No significant differences were observed in parameters of gender, average age, time from injury to surgery, Regan-Morrey type, cause of injury, laterality of injury and combined injury among the four groups (*p* > 0.05) (Table 1).

**Table 1:** Comparison of clinical characteristics of patients with ulna coronoid fractures among the four groups

	Mini plate group (*n* = 31)	Hollow screw group (*n* = 44)	Kirschner wire group (*n* = 62)	Steel wire suture group (*n* = 27)	*p*
Gender (male/female)	19/12	23/21	36/26	12/15	0.557
Average age (year)	38.8 ± 6.1	35.0 ± 6.9	35.5 ± 6.6	36.1 ± 6.4	0.076
Time between injury and surgery (d)	4.5 ± 1.1	4.8 ± 1.3	4.3 ± 1.3	4.4 ± 1.0	0.217
Regan-Morrey type					
II	21	29	34	17	0.56
III	10	15	28	10	
Cause of injury					
Fall	17	23	44	18	0.186
Traffic injury	14	21	18	9	
Laterality of injury					
Left side	12	19	38	14	0.134
Right side	19	25	24	13	
Combined injury					
Dislocation of elbow joint	21	27	44	23	0.200
Simple ulna coronoid fractures	10	17	18	4	

Notes: Measurement data or counting data were presented as mean ± standard deviation or number of cases; one-way analysis of variance (ANOVA) with replicate measurement or chi square test were used to compare multi-group data; a value of *p* < 0.05 was considered to be statistically significant

### Internal fixation with mini plate and hollow screw caused lower average operation time and blood loss than Kirschner wire and steel wire suture

**Fig. 1:**
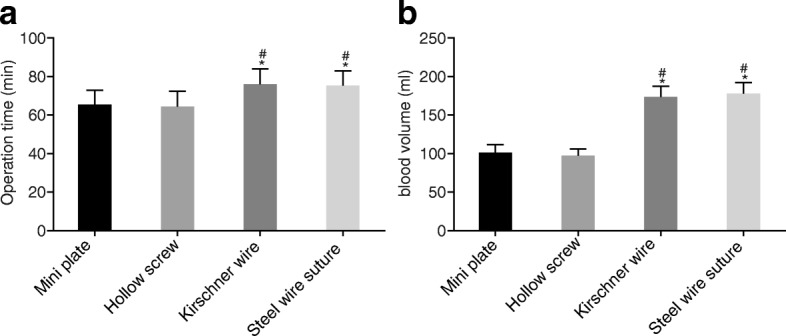
Internal fixation with mini plate and hollow screw decreases average operation time and blood loss in comparison to Kirschner wire and steel wire suture. **a** comparison of operative time of patients between four groups; **b** comparison of intraoperative blood loss of patients between four groups. *n* = 31 in the mini plate group; *n* = 44 in the hollow screw group; *n* = 62 in the Kirschnner wire group; *n* = 27 in the steel wire suture group; measurement data was presented as mean ± standard deviation; multi-group data was compared using single factor ANOVA; *, vs. the mini plate group, *p* < 0.05; ^#^, vs. the hollow screw group, *p* < 0.05

Following that, the different surgical conditions in four groups were compared. On comparing the operation time and intraoperative blood loss in the four groups, it was found that the mini plate group and the hollow screw group had significantly lower average operation time and average blood loss than the Kirschner wire group and the steel wire suture group (all *p* < 0.05). No significant differences were observed in the average operation time and average blood loss between the mini plate group and the hollow screw group (both *p* > 0.05). Similarly, no significant differences in average operation time and average blood loss were observed between the Kirschner wire group and the steel wire suture group, either (both *p* > 0.05) (Fig. 1). Altogether, patients undergoing internal fixation with mini plate and hollow screw had a shorter average operation time and lower blood loss than the patients with Kirschner wire and steel wire suture.

### The postoperative VAS pain scores are lower than the preoperative VAS pain scores

Next, preoperative pain and postoperative pain of patients in four groups were compared. VAS pain score was used to evaluate the preoperative and postoperative pain of patients in the four groups. It was found that the VAS pain scores 3 days after surgery and 1 month after surgery were significantly lower than the score prior to surgery in the four groups (all *p* < 0.05). On the 3rd day after surgery, the Kirschner wire group and the steel wire suture group had an obviously higher VAS pain scores compared to the mini plate group and the hollow screw group (all *p* < 0.05). However, no significant differences were observed in the intensity of pain of patients among the four groups 1 month after surgery (all *p* > 0.05) (Table 2). In summary, the postoperative pain was alleviated compared to the preoperative pain.

**Table 2:** Comparisons of preoperative and postoperative pain in the four groups

	Mini plate group (*n* = 31)	Hollow screw group (*n* = 44)	Kirschner wire group (*n* = 62)	Steel wire suture group (*n* = 27)
Before surgery	6.74 ± 0.77	6.41 ± 0.66	6.42 ± 0.62	6.63 ± 0.74
At 3 days after surgery	3.90 ± 0.75^*^	3.86 ± 0.46^*^	4.58 ± 0.67^*#&^	4.89 ± 0.51^*#&^
At 1 month after surgery	0.65 ± 0.49^*^	0.43 ± 0.50^*^	0.55 ± 0.50^*^	0.41 ± 0.50^*^

Notes: Measurement data were presented as mean ± standard deviation; one-way analysis of variance (ANOVA) with replicate measurement was used to compare data at different time points within-group, while multi-group data was compared using single factor ANOVA; n, number

*vs. the preoperative scores, *p* < 0.05; ^#^vs. the mini plate group, *p* < 0.05; ^&^vs. the hollow screw group, *p* < 0.05

### Mini plate and hollow screw show better elbow joint function than Kirschner wire and steel wire suture

Following that, the elbow joint function of patients in the four groups was compared. Broberg and Morrey elbow score was used to evaluate the postoperative function of elbow joint in the four groups. The results showed that at each given time point after surgery, the Kirschner wire group and the steel wire suture group had notably lower Broberg and Morrey elbow scores than the mini plate group and hollow screw group (all *p* < 0.05). However, the hollow screw group presented significantly higher Broberg and Morrey elbow scores than the mini plate group at 3 months, 6 months and 1 year after surgery time points (all *p* < 0.05) (Table 3).

**Table 3:** The function of elbow joint of patients with ulna coronoid fractures in the four groups

	Mini plate group (*n* = 31)	Hollow screw group (*n* = 44)	Kirschner wire group (*n* = 62)	Steel wire suture group (*n* = 27)
At 1 month after surgery	73.26 ± 10.49	78.45 ± 6.51	60.13 ± 12.42^*#^	60.59 ± 12.39^*#^
At 3 months after surgery	75.48 ± 10.62	85.84 ± 5.09^*^	63.94 ± 12.45^*#^	65.70 ± 10.81^*#^
At 6 months after surgery	80.48 ± 10.41	90.36 ± 5.16^*^	68.58 ± 12.54^*#^	70.41 ± 11.39^*#^
At 1 year after surgery	84.90 ± 11.23	94.39 ± 5.68^*^	73.48 ± 14.05^*#^	72.96 ± 13.74^*#^

Notes: Measurement data were presented as mean ± standard deviation; one-way analysis of variance (ANOVA) with replicate measurement was used to compare data at different time points within-group, while multi-group data was compared using single factor ANOVA

* vs. the mini plate group at the same time point, *p* < 0.05; ^#^vs. the hollow screw group at the same time point, *p* < 0.05

**Fig. 2:**
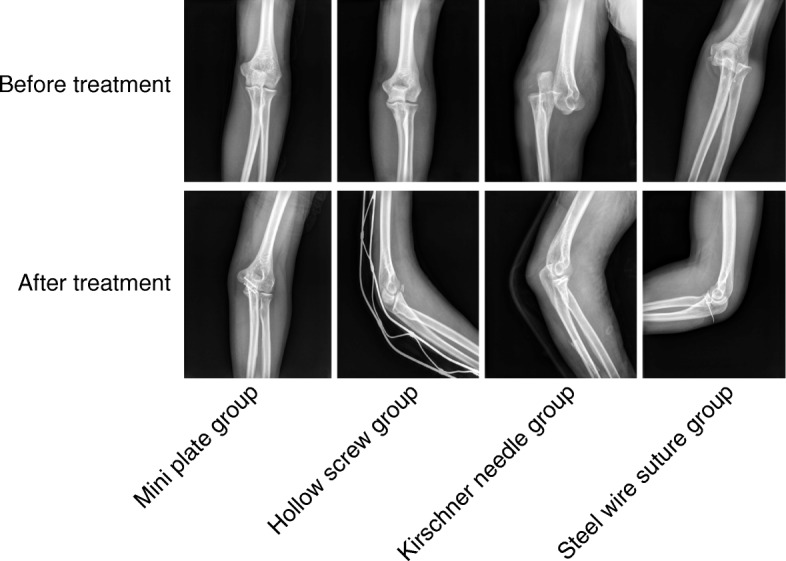
Mini plate and hollow screw displays better elbow joint function than Kirschner wire and steel wire suture

One year after surgery, the excellent and good functional outcomes of the mini plate group (54.8%) and the hollow screw group (90.9%) were significantly higher than those of the Kirschner wire group (29.0%) and the steel wire suture group (42.2%) (all *p* < 0.05), and the excellent and good functional outcomes of the hollow screw group was considerably higher compared to the outcomes of the mini plate group (*p* < 0.05). No significant difference was observed in the excellent and good functional outcomes between the Kirschner wire group and steel wire suture group (*p* > 0.05) (Table 4, Fig. 2). In conclusion, the elbow joint function was better in patients with Mini plate and hollow screw than the patients with Kirschner wire and steel wire suture.

**Table 4:** The surgical effect of patients with ulna coronoid fractures in the four groups

	Mini plate group (*n* = 31)	Hollow screw group (*n* = 44)	Kirschner wire group (*n* = 62)	Steel wire suture (*n* = 27)
Excellent	9	32	14	6
Good	8	8	4	0
Medium	12	4	36	17
Poor	2	0	8	4

Note: Counting data were presented as number of cases; chi square test was used to compare data at different time points within-group; a value of *p* < 0.05 was considered to be statistically significant

### Kirschner wire and steel wire suture cause higher incidence of complications and worse postoperative recovery

Finally, the postoperative recovery and complications were evaluated in the four groups. The findings indicated that the hollow screw group and the mini plate group had a significantly shorter fracture healing time and notably higher elbow flexion with better extension degree and forearm rotation degree than the Kirschner wire group and the steel wire suture group (all *p* < 0.05). No nonunion of fracture or delayed union was observed in the hollow screw group and the mini plate group. The hollow screw group had 2 cases of joint instability with 4.55% incidence of complications. The Kirschner wire group had 7 cases of delayed union, 5 cases of nonunion, 7 cases of failure of internal fixation, with 32.26% incidence of complications. The steel wire suture group had 2 cases of delayed union, 5 cases of heterotopic ossification, 4 cases of joint stiffness, with 37.04% incidence of complications. The patients in the Kirschner group and the steel wire suture group were at a higher risk for incidence of complications than the patients of the hollow screw group and mini plate group (Table 5). Therefore, higher incidence of complications and worse postoperative recovery were observable in patients with Kirschner wire and steel wire suture.

**Table 5:** Postoperative recovery and complications in the four groups

	Mini plate group (*n* = 31)	Hollow screw group (*n* = 44)	Kirschner wire group (*n* = 62)	Steel wire suture group (*n* = 27)
Fracture healing time (week)	10.1 ± 1.3	10.8 ± 1.0	13.4 ± 1.3^*#^	13.0 ± 1.6^*#^
elbow flexion and extension (°)	114.6 ± 6.5	115.4 ± 8.1	95.7 ± 8.1^*#^	94.4 ± 7.7^*#^
forearm rotation (°)	126.0 ± 7.3	127.7 ± 6.0	103.9 ± 4.2^*#^	102.5 ± 3.8^*#^
Incidence of complications	0 (0%)	2 (4.55%)	20 (32.26%)^*#^	10 (37.04%)^*#^

Notes: measurement data or counting data were presented as mean ± standard deviation or number of cases; one-way analysis of variance (ANOVA) with replicate measurement or chi square test were used to compare multi-group data

* vs. the mini plate group, *p* < 0.05; ^#^ vs. the hollow screw group, *p* < 0.05

## Discussion

Coronoid fractures have a low incidence and often occur in combination with other elbow joint injuries [6]. It is reported that the incidence of elbow dislocations is 5.21 per 100,000 people every year in America [21]. Previous evidence has shown that patients with proximal ulna fractures can restore a stable and functional elbow by means of open reduction and internal fixation [22]. Open reduction and internal fixation have been proven to be effective in treating anteromedial facet fracture of ulna coronoid by anatomical reduction and strong fixation [23]. In this study, it was found that internal fixation with mini plate and hollow screw had better efficacy than internal fixation with Kirschner wire and steel wire suture in treating Regan-Morrey type II and III ulna coronoid fractures.

The present study showed that internal fixation with mini plate and hollow screw had shorter average operation time, lower average blood loss, and less postoperative pain than internal fixation with Kirschner wire and steel wire suture while treating Regan-Morrey type II and III ulna coronoid fractures. A recent study showed that mini-locking plate fixation could be employed to treat Regan-Morrey type III coronoid fractures through elbow anterior approach, for satisfactory outcomes [24]. Another study also demonstrated that an anterior approach is effective for internal fixation of ulnar coronoid fractures because of minimal surgical dissection damage and clear visualization of ulnar coronoid fractures for reduction and internal fixation [25]. Moreover, among numerous surgical treatment techniques, mini plate and screw are superior options as they can stipulate rigid fixation important for progressive bone healing and early active finger motion [26]. In line with our study, Hanks et al. also confirmed that internal fixation of fracture of the coronoid process of the ulna is capable of restoring elbow stability [27]. Furthermore, a previous study has proven that mini-plate fixation as an effective treatment option for ulnar coronoid process fractures [28].

Additionally, our study also demonstrated that compared with internal fixation with Kirschner wire and steel wire suture, internal fixation with mini plate and hollow screw was more effective at restoring elbow joint function and lower incidence of complications after surgery. Another study also indicates that central tension plate internal fixation with sharp hook achieves excellent functional outcome in the treatment of intra-articular olecranon fracture as plate internal fixation is advantageous due to high union rate and low incidence of complications [29].

## Conclusion

To conclude, this study found that internal fixation with mini plate and hollow screw was more effective than internal fixation with Kirschner wire and steel wire suture for treating Regan-Morrey type II and III ulna coronoid fractures. Thus, this study supports some superior treatments for Regan-Morrey type II and III ulna coronoid fractures. However, some other internal fixation methods such as internal fixation of tension band wiring with steel wire should be considered and studied in the future.

## Abbreviations

ANOVA: Analysis of variance
VAS: Visual Analogue Scale
